# Diet and metabolism are back: The oldest known Islamic medical manuscript bridges the gap from ancient to modern gout management

**DOI:** 10.3892/mi.2023.114

**Published:** 2023-10-02

**Authors:** Styliani Geronikolou, George P. Chrousos, Demetrios A. Spandidos, Athanasios Diamantopoulos

**Affiliations:** 1 Biomedical Research Foundation of the Academy of Athens, 11527 Athens, Greece; 2 University Research Institute of Maternal and Child Health and Precision Medicine, National and Kapodistrian University of Athens, 11527 Athens, Greece; 3 Laboratory of Clinical Virology, Medical School, University of Crete, 71003 Heraklion, Greece; 4 Louros Foundation for the History of Medicine, 11528 Athens, Greece

**Keywords:** gout, purine-rich foods, dietary suggestions, old manuscript, al-Razi, history of urology, history of medicine

## Abstract

Gout is a chronic disease frequently associated with lifestyle; its prevalence is increasing in Western societies, as well as in the Middle East. Apart from its partial genetic features, diet accounts for 12% of the causality of the disease, while purine-rich foods contribute decisively to its development and evaluation. The influential Persian physician of the medieval ages, Abu Bakr Muhammad Ibn Zakariya al-Razi (or by his Latin name Rhazes; 860-925 AD), wrote a short book (20 chapters) entitled ‘*Treatise on gout*’. Rhazes adopted the Hippocratic humoralism, and suggested that the disease results from metabolic excess in the peripheral blood, presenting sex dimorphism and age-dependence. His therapeutic guidelines include a diet prescribed by a physician, the use of laxatives and emetics, counter-balancing excess or deficiency of bile or phlegm and analgesics, bloodletting, foot and steam baths, as well as salves and poultices as preventive measures. He appends differential dietary restrictions/suggestions for phlegmatic or choleric patients: Small rations and intake of good quality foods low in purine by 20% for phlegmatic and 28% for choleric patients, as well as the restriction of foods high in purine by 27% for phlegmatic and 22% for the choleric patients. Finally, the acidic to alkaloid food intake ratio suggested is 2/5 for choleric and 3/7 for phlegmatic patients. His suggested foods and drugs are vitamin C and B-rich complexes, thereby inhibiting the accumulation of tophi.

## Introduction

Gout is a chronic disease closely related to lifestyle, with an increasing prevalence in Western societies, as well as in the Middle East (1,2). It is denoted as ‘*the king of diseases and the disease of kings*’ (3). In other words, apart from its partial genetic vulnerability features (ATP binding cassette subfamily G member 2, glucokinase regulator, capping protein regulator and myosin 1 linker 1, PDZK domain-containing scaffolding protein, solute carrier family 2 member 9 and solute carrier family 16 member 9) (4), diet also plays a critical role in its manifestations (3,5). One of the main symptoms of gout is hyperuricemia (normal uric acid levels are 2.4-6.0 mg/dl in females and 3.4-7.0 mg/dl in males); thus, sex hormones play a decisive role in its appearance as stated in the Hippocratic Aphorisms (6-8). Its reported risk factors include metabolic syndrome, alcohol intake, dietary preferences (tailored to cultural and often racial diversities) (9), decreased physical activity, and hypertension (10). Diet accounts for 12% of the causality of the disease (11), while purine-rich foods contribute to its development and progression (12-14).

Gout is one of the earliest diseases described in Egyptian scrolls, in ancient Greek literature (Homer, Lucianus, Hippocrates, Galen), in Byzantine literature (Alexander of Tralles, John Chrysostomus, etc.) (15-19), as well as in Islamic medical manuscripts and/or translations (19-22). Dietary advice (restriction mostly) for patients with gout may also be found in ‘*Liber de podagra*’ authored by the Byzantine savant, Demetrios Pepagomenos, a 13th century AD manuscript (19,22) and in a Latin unpublished manuscript n: 98-6 of the 14-15th century AD (the manuscript was written in the 13th century AD, and transcribed in 15th century AD), entitled ‘*De arthetica passione*’ written by Antonio Guaniero da Pavia, and found in the Toledo Biblioteca Capitular (23).

The influential Persian physician of the medieval ages, Abu Bakr Muhammad Ibn Zakariya al-Razi (or by his Latin name Rhazes; 860-925 AD) wrote the ‘*Treatise on gout*’, probably, as a teaching handbook for his trainees. An unpublished Latin translation of this manuscript is preserved in the Bibliothèque de l' Arsènal, 1024 (71 SAL), fol 175 vo-181. Biblioteca Alexandrina, on the other hand, published the original 40 pages in 1199 AD (595 Η), transcribed by Aliy Sinān al-Sirāj al - Halabiy in a multilingual translation in 2003(21). The manuscript includes 20 chapters, where the titles have an interrogative style. A Greek translation of this manuscript was published in Athens in 2012(19).

Of note, Rhazes used to be proud of his Greek medical education, and his clinical point of view was humoralistic, involving the concept of four humors, as suggested by Hippocrates and further employed by Galen. In chapter IV, Rhazes clearly states that the disease results from metabolic excess in peripheral blood. This is shown by the following free translation (source language is Arabic): ‘*Any disorder in the blood consistency is a result of either yellow bile turning blood warm and dry, or phlegm that makes it gelatinous (thick) and phlegmatic. These two humours produce two types of gout: by choleric humour accumulation in the feet or by feet brimming with phlegm. Subjects with frail feet but lusty organs and overproduction of blood are exposed to a third type of gout, where plenty of blood attacks feet, inducing pain notwithstanding yellow bile or phlegm*’.

He notes that gout may be prevalent in adult males, rather than females before menopause (‘*because before menopause their bodies are humid, aborting unhealthy humours*’), in eunuchs (‘as their bodies are cold and weak’) or male prepuberts (‘*as their bodies are cold, humid and immature*’), as stated in chapters V-VII. In chapter X, he lists the treatment methods available in his time, highlighting that they supplement one another, as follows: i) Moderation of a restricted diet; ii) adopting physician-prescribed dietary guidelines; iii) the administration of laxatives and emetics; iv) bloodletting; v) application of water on feet; vi) steam baths; vii) administration of salves and poultices; viii) prevention of disease crises, ix) adopting drugs for counter-balancing excessive or excessed or deficient humors (anti-podagra) and analgesics.

All his thought is characterized by choleric vs. phlegmatic types of patients. Dietary preferences are health status moderators according to ancient Greek medicine (24). In chapters XI and XII, he provides detailed food guidelines for patients with gout, while he appends differential dietary restrictions/suggestions for phlegmatic or choleric patients.

Rhazes initiates his suggestions by emphasizing the need for small rations of high-quality foods and good hygiene standards. As ancient Greek doctors had written earlier, the undesirable effects of dietary excess may be treated with laxatives and emetics. Of note, these dietary dimorphic urges are presented as a supplement to the also humour-type targeted drug remedies. In a previous publication, the drug constituents and mode of administration (stated herein) is detailed (20).

## Preferred foods

The modest consumption of cereals rich in purines (e.g., rice) for both types of patients is proposed in this work of the 9th century; the same is also recommended in modern medical research (12,25). Rhazes notes that both types of patients should prefer spinach, beetroots and wheat in boiled form, as opposed to other cooking methods. Such patients would benefit from fruits with medium levels of sweetness, such as pears, grapes and figs, as opposed to other fruits and desserts, which are high in sugar. He explains that wheat or semolina bread and fish balls should be preferred. Soups containing beetroots or wheat are, modestly allowed.

More specifically, patients suffering from phlegmatic blood are advised to consume the following: i) Only small rations of lettuce, cucumber, gourd, lentils, mushrooms, cauliflower, cuscuta, eggplants, endives, cabbage and whey (these servings are used to substitute a common meat dish named *kisk*); ii) hens, chicken, pigeons, gulls, grouses, francolins cooked in a yeast sponge and stuffed with greens and spices, stewed or prepared with onions, vinegar, leeks and honey; iii) sprouts, young lambs fried with onions, chickpeas, almonds, eggs, and herbs or cumin mixed with vinegar and spices; iv) small fish or fish balls cooked with vinegar or served with amaracus (*Origanum majorana*) sauce; v) eggs, soft-boiled. On the other hand, choleric patients are advised to prefer small fried fish (in oil) apart from fish balls (in vinegar or amaracus sauce).

## Foods to abstain from

Both types of patients are recommended to avoid doves (šafānīn), cereals or pastries containing honey or syrup or having been fried. Greens are completely restricted, apart from those stated in the paragraph above. Almonds, raisins, pine seeds, dried dates, walnuts and peanuts are completely forbidden, and should only be consumed in very small rations. Servings containing beetroots and wheat (except boiled) and ‘red color dishes’ are classified as restricted by Rhazes. Rhazes clarifies that thick boiled or cooked eggs, beef, camel, sheep meat (*jupūr*) in general, and any meat and larger fish fried or salted and/or smoked and/or dried [either under the sun (*nakmasūd*) or in the oven], dairy products (except pastries containing dairy products, such as rice pudding), and almonds should be avoided by all gouty patients. Notably, all these foods have been classified to be highly or medium-rich in purines (13,26). Rhazes' remarks are unexpected, as purines were unimaginable entities in the 9th century, and his suggestions appear to be based on discerning observations. The importance of the purine concentration was evidenced a millennium later, in 2004 AD (13,14). Considering this, the restriction of foods with high purine concentrations is indicated as follows: 27% in phlegmatic and 22% in choleric patients. Accordingly, he suggests that foods low in purine to be restricted to 20% in phlegmatic and 28% in choleric patients.

More importantly, his suggested foods and drugs are vitamin C and B-rich complexes, thereby inhibiting the accumulation of tophi, in accordance with modern treatment approaches (4,27-29). Recent literature denotes that the DASH and Mediterranean diets, and the consumption of low-fat dairy products and cherries may contribute to a decrease in serum urate levels (9,30-35).

Metabolism converts anthocyanins, contained in many of the referred foods, to anti-inflammatory elements. Finally, the acidic to alkaloid food intake ratio suggested for choleric and phlegmatic patients should be 2/5 and 3/7 accordingly; these ratios, however, merit future validation by evidence obtained from randomized controlled trials.

This ancient manuscript is of importance for medical history, due to the information conveyed herein, which has not been previously communicated, at least to the best of our knowledge. Furthermore, contemporary research may be inspired by such manuscripts that incorporate dietary ancient observations to further evaluate medical/dietary knowledge in complex disease entities. Moreover, as the prevalence of gout in specific populations of distinct racial and ethnic backgrounds largely varies (9,35,36), the quantitative suggestions of Rhazes need to be validated beyond the Persian and Arabic populations.

## Data Availability

The datasets generated and/or analyzed during the current study are not publicly available due to the publishing rights of the publisher (Efporista), but are available from the corresponding author on reasonable request, or may be found in the e-ISBN: 978-618-80000-1-8 electronic book, or ISBN: 978-618-80000-0-1.
